# Effect of straw mulch and seeding rate on the harvest index, yield and water use efficiency of winter wheat

**DOI:** 10.1038/s41598-018-26615-x

**Published:** 2018-05-25

**Authors:** Changlu Hu, Cheng Zheng, Victor O. Sadras, Mao Ding, Xueyun Yang, Shulan Zhang

**Affiliations:** 1 State Key Laboratory of Soil Erosion and Dryland Farming on the Loess Plateau, Northwest A & F University, Yangling, 712100 Shaanxi China; 2 Key Laboratory of Plant Nutrition and the Agri-environment in Northwest China, Ministry of agriculture, College of Resources and Environment, Northwest A & F University, Yangling, 712100 Shaanxi China; 3 South Australian R & D Institute, Waite Campus, Adelaide, 5064 Australia

## Abstract

Straw mulching has been used to improve water use efficiency (WUE, yield per unit evapotranspiration) in the Loess Plateau of China, but the seeding rate may need to be adjusted from conventional practice. We tested the three-way interaction between seeding rate, soil management (conventional tillage and straw mulching) and seasons. Wheat yield ranged from 2851 to 6981 kg ha^−1^ and WUE from 5.3 to 16.2 kg ha^−1^ mm^−1^. Generally, soil water storage was higher and soil temperature was lower under straw mulching than under conventional practice. Evapotranspiration was higher under straw mulching. Yield was significantly affected by the interaction between straw mulching and season. Conventional practice showed significantly higher mean harvest index (HI), yield, and WUE than straw mulching in favorable seasons. However, yield was significantly higher under mulching than under conventional tillage in very dry season. Seeding rate had no effect on yield, but low seeding rate increased HI compared to high seeding rate. It is concluded that low seeding rate would be suitable for straw mulching.

## Introduction

In China Loess Plateau, dryland farming is practiced on about 12.8 million ha or 80% of its arable land^1^ where precipitation has declined at a rate of 1–2 mm y^−1^ in the last five decades^2,3^. With increasing population, the challenge is to increase food production with declining precipitation. Conservation tillage has been encouraged to conserve soil and water resources, and increase crop yield^4–10^. Winter wheat (*Triticum aestvum* L.) is widely grown in the Loess Plateau. About 40% of annual precipitation occurs from October through June when winter wheat is growing; hence, yield depends on both in-season precipitation and stored soil water at sowing, as in other systems^11,12^. Recent reviews have shown that the response of rainfed wheat yield to straw mulching in the Loess Plateau varies from −20 to 74%, and the response of water use efficiency (WUE, yield per unit evapotranspiration) varies from −9 to 96%^9,13^ compared with conventional practice, whereas harvest index is reduced under straw mulching^8,14–16^. These results indicate that there is an opportunity to further increase yield and WUE with practices to improve harvest index under straw mulching. We hypothesized that higher availability of soil water early in the growing season under mulch may favor early growth at the expense of growth at critical reproductive stages, hence affecting harvest index, yield, and WUE.

Seeding rate influences early biomass production^17^ and adjusting stand density to match water availability is common, for example, in the US corn-belt^18^. Generally, seeding rate of rain-fed wheat is adjusted to account for climate, soil, varieties and sowing date^19,20^. If straw mulch reduces grain yield by enhancing early growth and water use, then we can expect a three way interaction between soil management (mulch vs conventional), seeding rate and seasonal conditions. Few studies addressed this interaction. For rain-fed wheat in straw mulching systems or conservation tillage, the suitable seeding rates ranged widely between 111 and 470 seeds m^−2^ with different varieties^15,21–24^. For example, Sunderman^23^ concluded that seeding rates do not need adjustment under conservation tillage in various weather conditions. In wet and cold seasons, Kühling *et al*.^24^ documented no difference in wheat yield between two seeding rates (450 and 600 seeds m^−2^) across no-till and conventional practice in the south of Western Siberia. In the Loess Plateau under straw mulching, normal seeding rate (320 seeds m^−2^) and 25% higher rate returned similar yield^15^, but lower rates were not tested. Thus, our objective was to test the three way interaction between seeding rate, soil management and seasonal conditions, and its impact on grain yield and its components including harvest index, water use and water use efficiency of winter wheat. We hypothesized that soil water storage improved by straw mulching would stimulate tillering, early growth and water use, thus lower seeding rate under straw mulching could partially counteract the risk of lower grain yield and harvest index.

## Materials and Methods

### Site and experimental design and crop husbandry

Four-year field experiments were conducted at Wangdong village (35.14 N, 107.41 E, 1206 m above sea level), Shaanxi Province in the Loess Plateau from 2012 to 2016. Annual average temperature is 9.3 °C. The water table (50–80 m) is far below root zone and does not influence crop growth. Average annual precipitation is 578 mm, with 55% between July and September. Table 1 shows the actual rainfall in two periods, fallow and crop growing season, and the aridity index (precipitation divided by potential evapotranspiration) summarizing the seasonal conditions of this study.

**Table 1:** Winter wheat sowing and harvest date; precipitation during three periods: fallow (from wheat harvest at the end of June or early July to sowing in the late September or early October), in-crop season (from wheat sowing to harvest), and total (fallow + in-crop season), and aridity index at Wangdong village of Shaanxi Province.

Season	Sowing date	Harvest date	Precipitation (mm)			Aridity Index^†^		
			Fallow	In-crop season	Total	Fallow	In-crop season	Total
1	2012/9/21	2013/6/23	337	160	497	0.73	0.17	0.36
2	2013/9/29	2014/7/4	392	252	644	0.88	0.32	0.53
3	2014/10/2	2015/7/5	345	277	622	0.77	0.36	0.51
4	2015/9/25	2016/7/1	217	223	440	0.47	0.24	0.32

^†^Aridity index (AI) returns hyper-arid (AI < 0.05), arid (0.05 < AI < 0.2), semi-arid (0.2 < AI < 0.5), dry sub-humid (0.5 < AI < 0.65), and humid (AI > 0.65) environments.

The soil in this site is classified as an aridic and loamy, Cumulic Haplustoll^25^, or a calcisol (WRB 2014) with a silt loam texture developed from loess deposits. At the start of experiment, top soil (0–20 cm) had 14.4 g kg^−1^ organic matter, 0.95 g kg^−1^ total nitrogen, 12.9 mg kg^−1^ nitrate nitrogen, 1.8 mg kg^−1^ ammonium nitrogen, 19 mg kg^−1^ available phosphorus, 157 mg kg^−1^ available potassium and 1.21 g cm^−3^ bulk density.

The experiment included six treatments resulting from the factorial combination of two soil management practices, i.e. conventional tillage and straw mulching with reduced tillage, and three seeding rates. Low seeding rate (LS) was 225–320 seeds m^−2^ and represents 75% of recommended rate for conventional practice; medium seeding rate (MS) was 300–400 seeds m^−2^, and is the recommended rate for conventional practice^26^; high seeding rate (HS) was 375–480 seeds m^−2^ and represents 125% of MS. Conventional practice included about 10 cm high wheat stubble at harvest that was incorporated to the soil about one month later with tillage to ca. 20 cm depth, then leaving bare soil until sowing. At sowing, fertilizers were incorporated into the soil by rotary ploughing to about 15 cm depth. In the mulch treatment, we established 0.6 kg dry stubble m^−2^, after wheat harvest on July 2012 and maintained ground cover without tillage until sowing. At sowing, all wheat straw was temporarily removed when fertilizers were incorporated into the soil as conventional practice, and wheat was sowed manually. Then wheat straw returned to the plots evenly after correcting weight as it deteriorated during the time. Sowing rate and soil management treatments were arranged in a randomized block design with four replicates. Plot size was 20 m^2^ (4 × 5 m) including 20 rows spaced at 25 cm.

Winter wheat (cv Changhan 58) was sown in late September or early October and harvested in late June or early July, as shown in Table 1. Applied fertilizers were 150 kg N ha^−1^ as urea and 75 kg P_2_O_5_ ha^−1^ as triple superphosphate in the first three seasons, and 120 kg N ha^−1^ and 75 kg P_2_O_5_ ha^−1^ in the last season. Nitrogen rates were based on soil nitrate nitrogen content before sowing (45 kg N ha^−1^ in the topsoil, 0–20 cm) and N rate experiment at the same site showing no yield difference between 120 and 180 kg N ha^−1^. Weeds and diseases were managed as recommended locally. Additionally, in the fourth season, we found some abnormal stems caused by wireworms (Elateridae) at GS39^27^. Thereafter, pests were immediately controlled with cyhalothrin.

### Sampling and measurements

Wheat shoot population was recorded at emergence (GS15), tillering in winter (GS25), jointing (GS35), and heading (GS59). At physiological maturity (GS92), number of culms or ears were counted from 0.5 m^2^ in each plot, it should be noted that the number of culms not bearing a spike were not included. At maturity, grain yield (12.5% moisture content), biomass, kernel-weight, the number of ears, and grains per ear were measured in each plot, and harvest index (HI) was calculated.

Water content in the soil profile (0–3 m) was measured gravimetrically (one profile per plot) at sowing, anthesis (GS65) and harvest (GS92). Soil temperature was measured for about one week centred at three growth stages (GS30, GS35 and GS65) in 2013–2014, 2014–2015 and 2015–2016. Since the second wheat season, daily soil temperature was derived by average of measurements at 8:00 a.m., 2:00 p.m. and 8:00 p.m. using bent-stem soil thermometers installed at 5, 10, 15 and 20 cm depth in three replicated plots for the medium seeding rate under both conventional and straw mulching treatments.

Precipitation was measured with a data logger (CR1000, Campbell Sci.) located about 1 km away from the experimental field. The sensor was factory calibrated before installation.

### Calculations and statistical analyses

Crop evapotranspiration (ET) was estimated from water balance equation;

1 $${\rm{ET}}={\rm{P}}+{\rm{\Delta }}{\rm{W}}$$

where P is precipitation and ΔW change in soil water from sowing to harvest. We assumed negligible runoff, based on the flatness of the plots, and negligible drainage as precipitation rarely wets the soil below 2 m, whereas ΔW was measured down to 3 m. Water use efficiency was calculated as grain yield divided by ET^9^.

Repeated measures ANOVA (general linear model) was used to evaluate the effects of season, soil management, seeding rate and their interactions on crop traits (yield, biomass, HI, ear number, grain number, kernel weight, ET, WUE) and soil water storage. In this model, the test seasons were referred to as the within-subjects factors, and the soil management and seeding rate were referred to as the between-subjects factors. A Greenhouse-Geisser correction was applied to the *F*-ratio, if the sphericity assumption was not met. Soil temperature was analysed with *t*-test to compare conventional practice and straw mulching. For each season, one-way ANOVA was performed to evaluate effects of seeding rate on crop traits and soil water. When *F*-values were significant, multiple comparisons of mean values were performed using the least significant difference method (LSD) at the 0.05 probability level. All statistical analysis was performed through SPSS software.

## Results

### Weather

**Figure 1:**
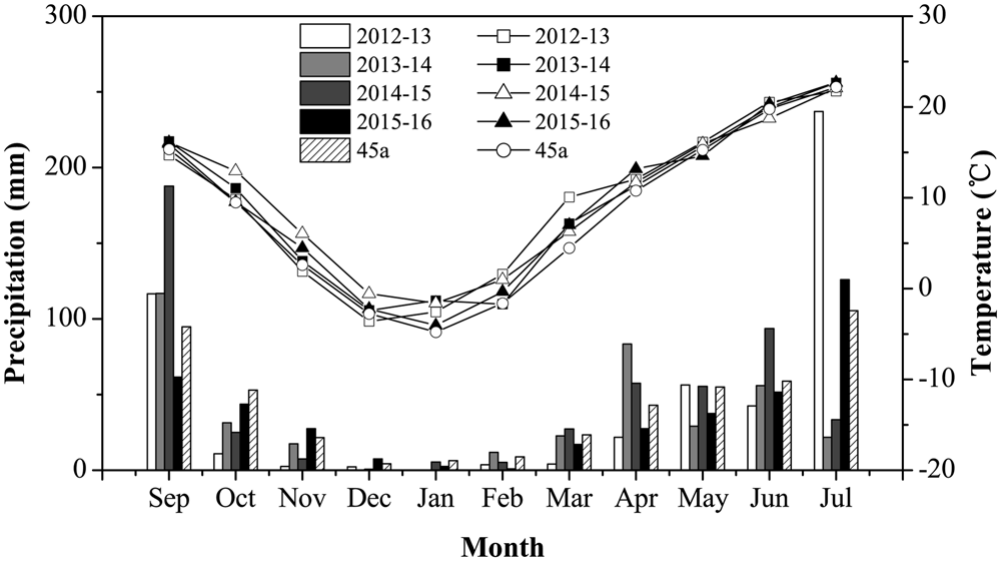
Monthly precipitation, monthly mean temperature, and the 45 years average (45a) during the growing period of winter wheat (September to July) in four seasons at Wangdong village of Shaanxi Province.

Figure 1 shows precipitation and air temperature during the four seasons. In four years, air temperatures during the growing season were generally similar to the long-term. Frost occurred in early April in 2013 and 2015 (beginning of jointing stage), which affected wheat stand and yield components. Additionally, hail occurred on grain filling in 2015 (May 30, 2015).

The long-term fallow, in-crop and total precipitation were 304 mm, 274 mm and 578 mm, respectively. Total precipitation was below the long-term mean in the first season, mainly due to the very low in-crop precipitation of 160 mm. The fourth season also showed lower total precipitation (440 mm) relative to the long-term, but precipitation was more evenly distributed during wheat growth. Both the second (644 mm) and third (622 mm) seasons were wetter than the long-term mean, (Table 1). Fallow rainfall accounted for 68% (2012–13), 61% (2013–14), 55% (2014–15) and 49% (2015–16) of annual precipitation.

### Soil water and thermal conditions

**Figure 2:**
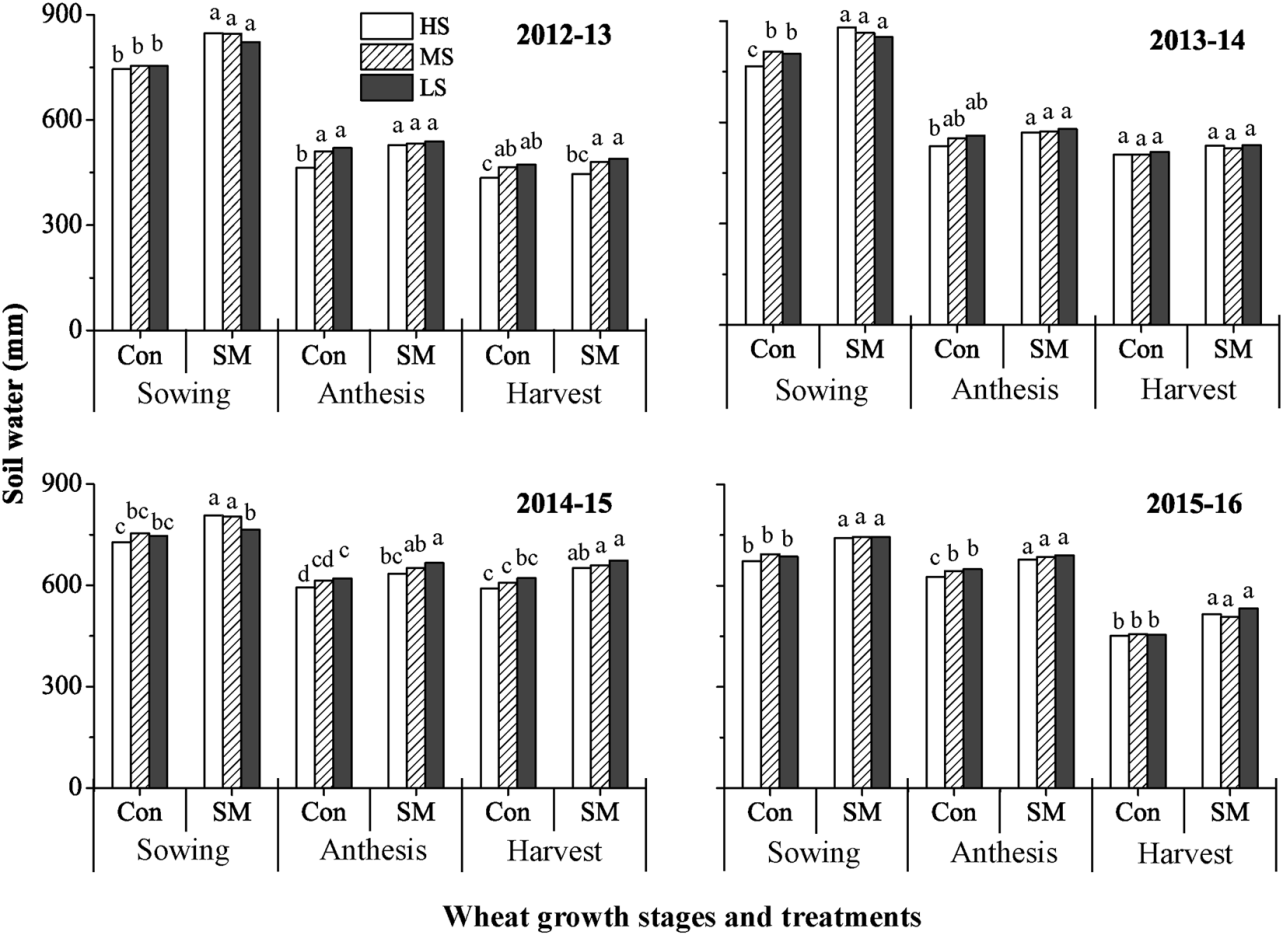
Soil water storage (0–3 m) at sowing, anthesis, and harvest in wheat crops in a factorial experiment combining three seeding rates, two soil management practices and four seasons at Wangdong village of Shaanxi Province. Seeding rates are high (HS), medium (MS) and low (LS) and soil management practices are conventional tillage (Con) and straw mulching (SM). Different letters mean significant difference between treatments at a given stage each year (*P* < 0.05, n = 4).

Table 2 shows ANOVA for soil water at sowing, anthesis and harvest as affected by season, straw mulching, seeding rate and their interactions. Soil water at sowing varied seasonally, and was significantly higher under mulch than under conventional treatment (Table 2, Fig. 2). Straw mulching increased soil water storage at sowing by 88 mm in the first season, by 73 mm in the second season, by 49 mm in the third season and by 60 mm in the fourth season in comparison to conventional tillage. At anthesis and harvest, straw mulching showed higher soil water content than conventional practice in the third and fourth seasons, but there was no difference in the first and second seasons.

**Table 2:** *P*-values from ANOVA testing effect of season, soil management (conventional tillage and straw mulching), seeding rate and their interactions on wheat grain yield, shoot biomass, harvest index (HI), yield components, evapotranspiration (ET), water use efficiency (WUE, yield per unit ET) and soil water storage at sowing, anthesis and harvest.

Item^†^	Yield	Biomass	HI	Ears No.	No. of grains	kernel weight	ET	WUE	W_1_^‡^	W_2_	W_3_
s	0.000	0.000	0.000	0.000	0.000	0.000	0.000	0.000	0.000	0.000	0.000
sm	0.001	0.131	0.102	0.025	0.003	0.747	0.021	0.010	0.000	0.001	0.000
sr	0.537	0.611	0.049	0.124	0.619	0.504	0.028	0.130	0.230	0.006	0.000
s × sm	0.004	0.012	0.019	0.532	0.001	0.440	0.001	0.433	0.007	0.578	0.003
s × sr	0.877	0.854	0.042	0.973	1.000	0.208	0.586	0.844	0.191	0.316	0.146
sm × sr	0.636	0.635	0.394	0.459	0.407	0.636	0.490	0.697	0.539	0.754	0.108
s × sm × sr	0.665	0.506	0.010	0.908	0.390	0.190	0.351	0.976	0.126	0.963	0.773

^†^s, season; sm, soil management; sr, seeding rate. ^‡^W_1_, W_2_ and W_3_ represent the soil water storage at sowing, anthesis and harvest, respectively.

Seeding rate significantly affected soil water storage, with high seeding rate reducing soil water content at anthesis and harvest in comparison to medium and low seeding rates (Table 2). Under conventional treatment, soil water storage was lower at the high seeding rate than the low seeding rate at anthesis except for the second season (2013–14) (Fig. 2). However, under straw mulching soil water storage was mostly at the same level for the three seeding rates at anthesis except for the third season (2014–15) (Fig. 2). In both conventional treatment or straw mulching, high seeding rate considerably decreased soil water at harvest only in the first season (Fig. 2).

**Figure 3:**
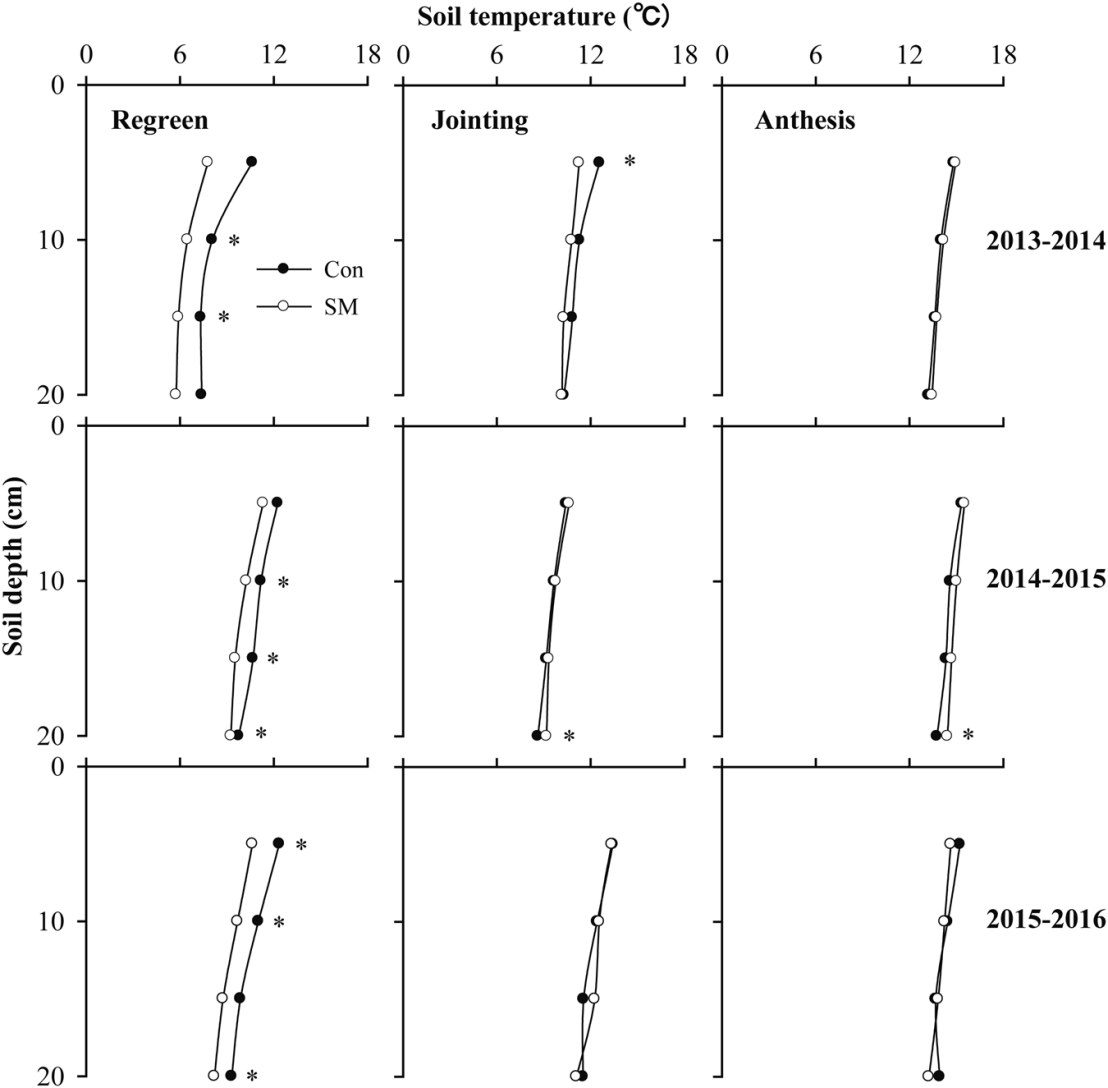
Soil temperature (0–20 cm) during the wheat growing period in 2013–2014, 2014–2015 and 2015–2016 at medium seeding rate. Conventional tillage (Con) and straw mulching (SM) treatments are compared at regreen, jointing and anthesis stages. *Represents significant difference between two treatments (*P* < 0.05, n = 3).

Top-soil temperature (5 cm) under mulching was 2.8 °C (2013–14), 0.9 °C (2014–15) and 1.7 °C (2015–16) cooler than under conventional practice at re-green stage (GS30), and 1.3 °C cooler at jointing (GS35) in 2013–2014 (Fig. 3). At other stages, soil temperatures were similar between the two treatments (Fig. 3).

### Wheat shoot population dynamics

**Figure 4:**
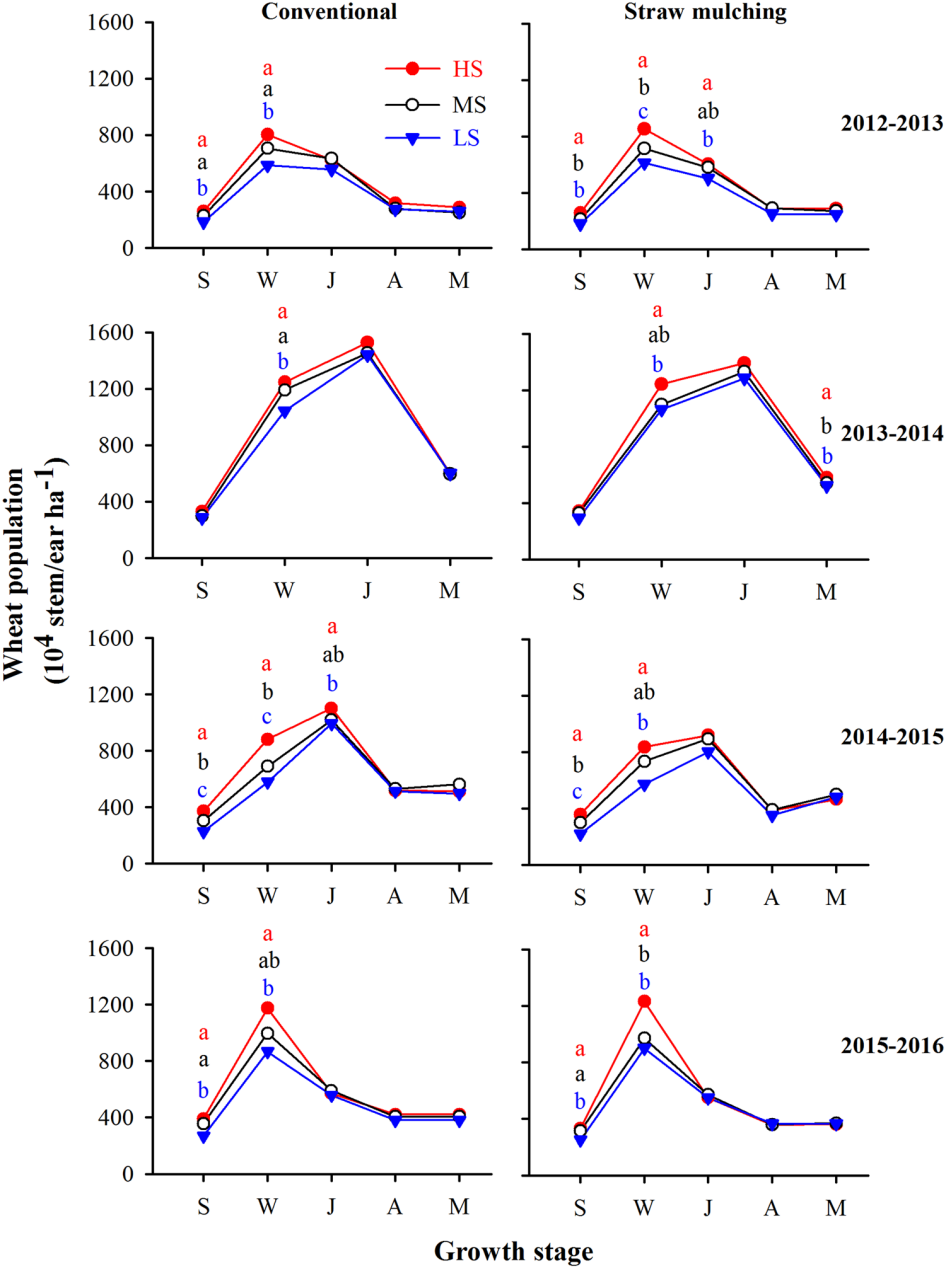
Dynamics of wheat shoots in a factorial experiment combining three seeding rates (high, HS; medium, MS; low, LS), two soil management practices (conventional tillage, straw mulching) and four seasons at Wangdong village of Shaanxi Province. Crop stages are S, Seedling; W, Winter; J, Jointing; A, Anthesis; M, Maturity. Different letters indicate significant differences between seeding rates (*P* < 0.05, n = 4).

Wheat shoot population was higher in the second than in the first, third and fourth seasons (Fig. 4). Seeding rate showed season- and practice-dependent effects on population size. Under conventional practice in the first and fourth seasons, shoot population was higher at high and medium than at low seeding rate at seedling and wintering stages; in the second season, high and medium seeding rates showed higher populations than low seeding rates at wintering stage; in the third season, high seeding rate presented higher populations than medium and low seeding rate from seedling to jointing (Fig. 4 left panels). Under straw mulching, high seeding rate presented higher shoot populations than medium and low seeding rate from seedling to jointing in 2012–2013, at wintering and maturity stages in 2013–2014, and at seedling and wintering stages in 2014–2015 and 2015–2016 (Fig. 4 right panels). No difference was observed at other stages.

There were no significant differences in shoot populations between conventional and straw mulching treatments at any seeding rate in the first and fourth seasons. However compared with conventional practice, populations with straw mulching were lower for all seeding rates at jointing in the second and third seasons, and for medium and low seeding rates at maturity in the second season (Fig. 4, Table 3).

**Table 3:** Yield, yield components, ET and WUE of wheat crops in a factorial experiment combining three seeding rates, two soil management practices and four seasons at Wangdong village of Shaanxi Province.

Season	Soil manage-ment^†^	Seeding rate^‡^	Yield (kg ha^−1^)	Biomass (kg ha^−1^)	HI	Ears No. (10^4^ ha^−1^)	No. of grains (grains m^−2^)	Kernel weight (mg)	ET (mm)	WUE (kg ha^−1^ mm^−1^)
2012–13	Con	HS	3113b^*^	7783a	0.40b	287a	8481ab	44.4a	478bcd	6.58a
		MS	2962b	7295a	0.41b	251a	7715b	44.4a	462 cd	6.31ab
		LS	3049b	7149a	0.43b	259a	7985b	45.0a	438d	6.76a
	SM	HS	2851b	7440a	0.39b	288 a	8332b	45.9a	543a	5.28b
		MS	3709 a	7530a	0.49a	273a	9328a	45.9a	527ab	7.27a
		LS	3317ab	7516a	0.44ab	258a	8413ab	45.8a	500abc	6.79a
2013–14	Con	HS	6298ab	16499ab	0.38ab	597a	21924a	35.1a	543b	11.67a
		MS	6533a	16895ab	0.39ab	596a	20296abc	35.8a	567ab	11.54a
		LS	6342ab	15968b	0.40a	600a	21544ab	34.3a	555ab	11.55a
	SM	HS	6157ab	16720ab	0.37bc	581a	19667bc	36.4a	602a	9.27b
		MS	6060bc	17031a	0.36c	542b	20270abc	34.6a	594a	9.42b
		LS	5664c	15973ab	0.35c	523b	18443c	35.8a	571ab	9.86b
2014–15	Con	HS	4180a	13284a	0.33a	512a	15385a	36.9b	414ab	10.83a
		MS	4179a	12878ab	0.32a	506a	13907ab	41.6a	412ab	10.23a
		LS	4174a	11842abc	0.35a	473a	13878ab	41.4a	390b	10.48a
	SM	HS	3207b	10538c	0.30a	476a	11078bc	39.6ab	433a	7.47b
		MS	3023b	10104c	0.30a	461a	10260c	40.0ab	431a	7.23b
		LS	3126b	10739bc	0.33a	452a	10803c	39.0ab	398b	7.80b
2015–16	Con	HS	6936a	13888a	0.53b	421a	17090a	50.9a	454a	16.24a
		MS	6981a	12467ab	0.56a	407a	15898ab	51.2a	455a	15.35ab
		LS	6881a	12237b	0.56a	380a	15649ab	51.6a	455a	15.17ab
	SM	HS	6496a	12805ab	0.51c	364a	14615bc	50.8a	459a	13.62b
		MS	6414a	12769ab	0.53b	369a	14635bc	50.9a	471a	13.49b
		LS	6506a	12060b	0.51c	366a	13764c	51.9a	454a	14.62ab

^†^Con, conventional tillage; SM, straw mulching. ^‡^HS, high seeding rate; MS, medium seeding rate; LS, low seeding rate. *The different lowercase letters represent significant difference between all treatments each season (*P* < 0.05) (n = 4).

### Yield and yield components

Wheat yield and its components are shown in Table 3, and Table 2 shows ANOVA. ANOVA indicated significant seasonal effects on grain yield as follows: season 4> season 2> season 3> season 1. Seasons also affected biomass, HI and yield components (Table 2). Across four seasons conventional practice had significantly higher grain yield, ear number and grain number compared with straw mulching. However, mulching significantly interacted with seasons; for example, in the very dry season 2012–2013 mulching increased HI and yield compared with conventional practice (Table 3). The high seeding rate showed distinctly lower HI than the low seeding rate (Table 2). Season, soil management and seeding rate also had significant interaction effect on HI.

Under conventional practice seeding rate had no effect on grain yield for all seasons, and on biomass and harvest index in first three seasons (Table 3). However, high seeding rate increased biomass and reduced HI compared with low seeding rate in the fourth season. Under straw mulching, medium seeding rate presented notably higher HI and yield than high seeding rate in the first season, but seeding rate did not affect HI and yield in the other three seasons, except for a higher HI in the medium seeding rate compared to both high and low seeding rate in fourth season (Table 3). With few exceptions, seeding rate had no effect on grain number and kernel weight under conventional practice or straw mulching in all tested seasons (Table 3). For example, high seeding rate had lower kernel weight than medium and low seeding rate in third season under conventional practice. Under straw mulching, high seeding rate had significantly higher ear number than medium and low seeding rates in the second season; additionally medium seeding rate had significantly higher grain number than high seeding rate in first season.

### Water use and water use efficiency

ANOVA indicated significant seasonal effects on ET and WUE (Table 2). Straw mulching generally increased ET and reduced WUE with respect to conventional practice. The interaction between straw mulching and season affected ET, but not WUE. Evapotranspiration was higher in crops with high and medium seeding rates compared to their low seeding rate counterparts (Table 2).

Under conventional practice ET and WUE were statistically similar among seeding rates in all seasons (Table 3). Under straw mulching, however, in very dry season (2012–2013) the WUE was significantly lower at high seeding rate (5.28 kg ha^−1^ mm^−1^) than at medium (7.27 kg ha^−1^ mm^−1^) and low (6.79 kg ha^−1^ mm^−1^) seeding rates (Table 3).

## Discussion

Owing to drier conditions and frost in 2012–2013 and frost and hail in 2014–2015, yield and WUE were lower in the first and third than in the second and fourth seasons. In addition, grain yield was higher in the fourth season than in the second season despite higher soil water storage at sowing and higher in-crop precipitation in the second season. Higher grain yield was mainly connected to higher kernel weight, which was partially related to precipitation distribution (10 mm more post-anthesis) and 5 days longer duration of grain filling in the fourth season than in the second season. Additionally, soil water storage at anthesis was higher in the fourth season than in the second season, and the opposite was true at harvest, which meant post-anthesis water use was more in the fourth than in the second season. The higher water availability after anthesis contributing higher yield has been discussed previously^9^.

Straw mulching reduced harvest index, grain yield, and WUE compared to conventional practice in three out of four seasons (wet and favourable condition), but straw mulching increased both harvest index and yield in the very dry season 2012–13. Amato *et al*.^28^ reported a similar interaction in a long-term experiment whereby wheat under no-tillage out-yielded its counterpart under conventional tillage under water stress but yield differences were reversed in wetter conditions. The global analysis of Pittelkow *et al*.^5,6^ showed that no-tillage in combination with residue retention in rainfed systems reduced crop yield in humid climates, but enhanced yield in dry climates. This study did not support our hypothesis that higher soil water availability early in the season under mulch may stimulate tillering and favour early growth at the expense of growth at critical reproductive stages, hence reducing harvest index and grain yield. Instead, we found that population size was generally similar between conventional treatment and straw mulching, and soil water content was consistently higher under straw mulching during the growing season. The reduction in harvest index, grain yield and WUE with mulching in those three seasons was primarily associated with fewer grains per area caused by lower ear numbers. Two factors may have contributed to fewer ears per unit area under mulch: cooler soil during tiller development in spring as found in our study (Fig. 3) and others under same practice^8^ or no-tillage^24^, and pest damage as noticed in our experiment and reported by others^29,30^. Thus, controlling pests should require more attention under straw mulching to capture the benefits of this practice, especially in wet seasons.

Seeding rate did not affect wheat yield and WUE when averaged across two soil managements over four seasons. Gao *et al*.^15^ found it was not necessary to increase seeding rate over the recommended level during three dry seasons under straw mulching in the same region. Our results highlighted that the higher depletion of water under high seeding rate especially during vegetative stage reduced harvest index compared with that under low seeding rate. Moreover, low seeding rate significantly reduced ET and tended to increase WUE. These results indicate the recommended seeding rate was still higher than needed. Thus, the low seeding rate tested in our study seems suitable for both conventional and straw mulching conditions. In addition, Sunderman^23^ has reported seeding rates at 75%, 100% and 125% of the recommended rate for the conventional practice produced similar wheat yield under no tillage, and the seeding rate of 75% of recommended level was suitable for various weather conditions. Our straw mulching treatment fully captured the effect of soil cover, and partially accounted for the reduced soil disturbance in farm-scale no-till systems. With this caveat, the present study showed that reducing seeding rate to 75% of the recommended rate would modulate soil water use and stabilise harvest index to improve the benefits of straw mulching under the prevalent conditions of the Loess Plateau.
